# Performance of an ultra-sensitive *Plasmodium falciparum* HRP2-based rapid diagnostic test with recombinant HRP2, culture parasites, and archived whole blood samples

**DOI:** 10.1186/s12936-018-2268-7

**Published:** 2018-03-17

**Authors:** Smita Das, Roger B. Peck, Rebecca Barney, Ihn Kyung Jang, Maria Kahn, Meilin Zhu, Gonzalo J. Domingo

**Affiliations:** Diagnostics Program, PATH, Seattle, WA USA

**Keywords:** Malaria, Histidine-rich protein 2, Rapid diagnostic test

## Abstract

**Background:**

As malaria endemic countries shift from control to elimination, the proportion of low density *Plasmodium falciparum* infections increases. Current field diagnostic tools, such as microscopy and rapid diagnostic tests (RDT), with detection limits of approximately 100–200 parasites/µL (p/µL) and 800–1000 pg/mL histidine-rich protein 2 (HRP2), respectively, are unable to detect these infections. A novel ultra-sensitive HRP2-based Alere™ Malaria Ag P.f RDT (uRDT) was evaluated in laboratory conditions to define the test’s performance against recombinant HRP2 and native cultured parasites.

**Results:**

The uRDT detected dilutions of *P. falciparum* recombinant GST-W2 and FliS-W2, as well as cultured W2 and ITG, diluted in whole blood down to 10–40 pg/mL HRP2, depending on the protein tested. uRDT specificity was 100% against 123 archived frozen whole blood samples. Rapid test cross-reactivity with HRP3 was investigated using *pfhrp2* gene deletion strains D10 and Dd2, *pfhrp3* gene deletion strain HB3, and controls *pfhrp2* and *pfhrp3* double deletion strain 3BD5 and *pfhrp2* and *pfhrp3* competent strain ITG. The commercial Standard Diagnostics, Inc. BIOLINE Malaria Ag P.f RDT (SD-RDT) and uRDT detected *pfhrp2* positive strains down to 49 and 3.13 p/µL, respectively. The *pfhrp2* deletion strains were detected down to 98 p/µL by both tests.

**Conclusion:**

The performance of the uRDT was variable depending on the protein, but overall showed a greater than 10-fold improvement over the SD-RDT. The uRDT also exhibited excellent specificity and showed the same cross-reactivity with HRP3 as the SD-RDT. Together, the results support the uRDT as a more sensitive HRP2 test that could be a potentially effective tool in elimination campaigns. Further clinical evaluations for this purpose are merited.

## Background

Since 2000, global malaria incidence and mortality have decreased dramatically, by 41 and 67%, respectively [1]. The improvements in disease burden, particularly for *Plasmodium falciparum* malaria, have been attributed largely to effective control programmes that include vector control, artemisinin-based combination therapy (ACT), rapid diagnostic tests (RDTs), and intermittent preventive treatment in pregnancy (IPTp) [1]. The success of control strategies has shifted many countries to pre-elimination; between 2000 and 2015, 17 countries reached elimination, of which 6 countries were declared as malaria-free [1]. However, malaria remains a major global health problem, with approximately 3.2 billion people across 91 countries at risk of infection [2]. Key challenges, including drug and insecticide resistance and parasite and mosquito plasticity, threaten the gains made in malaria risk reduction [2].

In low prevalence regions, subpatent *P. falciparum* infections have become an issue of increasing concern [2–6]. These subpatent carriers have been estimated to contribute to 20–50% of human-to-mosquito transmission, emphasizing the role of this reservoir population in continued transmission [6]. Furthermore, a majority of these parasite carriers have infections undetectable by microscopy and RDT, both of which have similar detection limits in terms of parasites per µL (p/µL), at approximately 100–200 p/µL [5, 7–10]. The poor performance of commercially available RDTs in terms of sensitivity for low density parasite infections limits their utility in elimination strategies. In many settings, molecular nucleic acid–based tests become the most reliable tests for understanding the levels of infection in a population [7, 8, 11, 12]. While nucleic acid-based testing affords the benefits of sensitivity, it is costly and often challenging to implement both from technical and logistical perspectives for many malaria programmes.

Modelling of mass screen-and-treat strategies suggests that a RDT with limits-of-detection (LOD) on the order of  2–20 p/μL expands the entomological inoculation rate (EIR) at which transmission could be interrupted from less than 1–4 [6]. Likewise, a more sensitive RDT could be used to trigger mass drug administration (MDA), as long as its ability to predict malaria prevalence approximates that of nucleic acid tests [6]. A diagnostic test for malaria with a 10- or 100-fold improvement in limit of detection (LOD) over microscopy or current RDTs that is simple to use and can be used at the point-of-care could be a useful tool for elimination strategies. RDTs targeting the *P. falciparum*-specific antigen histidine-rich protein 2 (HRP2) remain the most sensitive and robust tests for diagnosis of *P. falciparum* malaria, in part due to poorer LODs of tests for the other commonly used antigen, lactate dehydrogenase (LDH), as well as HRP2 accumulation during infection [13].

A novel ultra-sensitive Alere™ Malaria Ag P.f RDT (uRDT) has been recently developed. The uRDT takes advantage of the immunochromatographic cassette platform, whole blood volume requirements (5 µL), and slightly longer time to results as the current Standard Diagnostics, Inc. BIOLINE Malaria Ag P.f RDT (SD-RDT) Because the general test format of the uRDT is similar to the SD-RDT and most field teams are well versed in performing the SD-RDT, the uRDT fulfills many of the assured criteria and is additionally portable, stable, and functional in resource-limited areas [10, 14].

In this study, the uRDT detection threshold and test performance were investigated using different *P. falciparum* HRP2 types and parasite culture strains.

## Methods

### Rapid diagnostic tests (RDTs)

**Fig. 1:**
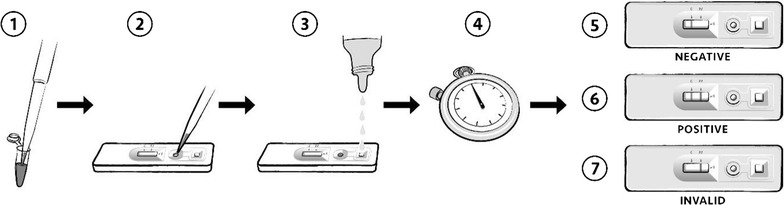
uRDT work flow. (1) 5 μL of sample is collected and (2) delivered to the sample well in the test cassette, followed by (3) the addition of four drops of assay buffer. (4) The test is allowed to incubate and the uRDT results are interpreted after 20 min while the SD-RDT results are interpreted after 15 min. The presence of a line of any intensity is considered for interpretation. (5) Tests with only a control line are interpreted as negative. (6) Tests with both control and test lines are interpreted as positive. (7) The absence of a control line indicates the test is invalid and should be repeated

All whole blood specimens were tested for *P. falciparum* using the commercially available Standard Diagnostics Inc. BIOLINE Malaria Ag P.f RDT (SD-RDT) (Reference number: 05FK50, Test lot number: 05CDA051A, Sub: A, Diluent lot number: 05BDDA102, Manufacture date: 2015.08.23, Expiry date: 2017.08.22; Republic of Korea) and Alere™ Malaria Ag P.f RDT (uRDT) (Reference number: 05FK140, Test lot number: 05LDB001A, Sub: A, Diluent lot number: 05BDDA145, Manufacture date: 2015.02.25, Expiry date: 2017.02.25; Republic of Korea). The uRDT, like the SD-RDT, is an immunochromatographic membrane strip test, but differs in that it uses biotinylated and carboxyl-modified latex fragment antibodies (FAbs) and polystreptavidin bound to the test line to detect *P. falciparum*-specific HRP2 in whole blood. The workflow of the uRDT is comparable to that of the RDT (Fig. 1). The test requires 5 µL of whole blood specimen. The blood is applied to the sample port in the test followed by application of 4 drops of assay diluent. Twenty minutes after application of the specimen, the result is interpreted from the result window. A positive control line (red) with a positive test line (blue/black) is interpreted as a positive result, and a positive control line and negative test line is interpreted as a negative result. Any test without a signal on the control line is considered an invalid test.

### Benchmarking

Benchmark testing was performed to define the test LOD for HRP2. Two *P. falciparum* HRP2 recombinant proteins, GST-W2 (rGST-W2) (Microcoat Biotechnologie GmbH, Bernried am Starnberger See, Germany) and FliS-W2 (rFliS-W2) (Alere™, San Diego, California), and two *P. falciparum* culture strains, W2 (ZeptoMetrix Corporation, Buffalo, New York) and ITG (BEI Resources, Manassas, Virginia), were selected for testing. All HRP2 types were stored at  −80 °C and thawed on ice during testing. The rGST-W2 and rFliS-W2 proteins were both supplied at 100 µg/mL. Each recombinant protein was diluted in negative whole blood (Interstate Blood Bank, Inc., Memphis, Tennessee; K3-EDTA anticoagulant) to 500 and 250 pg/mL, and tested with the Malaria Antigen (HRP2) CELISA (Catalog number: KM_2_, batch number: KM269; Cellabs, Sydney, NSW, Australia). Concentrations calculated with CELISA approximated the respective concentrations (rGST-W2: 518 and 219 pg/mL; rFliS-W2: 492 and 240 pg/mL). Subsequent dilutions of recombinant protein were prepared using the reported concentration from the stock protein.

For the *P. falciparum* native proteins, W2 culture was provided in prepared aliquots with reported HRP2 concentrations of 800 and 253 pg/mL. These concentrations and an additional dilution of the 800 pg/mL concentration in negative whole blood to 250 pg/mL were tested with CELISA. Concentrations calculated with CELISA were 588, 104 and 172 pg/mL, respectively. Subsequent dilutions of W2 culture were prepared using the concentrations calculated from CELISA. ITG was cultured in type O+ human erythrocytes diluted at 4% hematocrit in RPMI-1640 media (KD Medical, Columbia, Maryland) supplemented with 25 mM HEPES (Gibco, Gaithersburg, Maryland), sodium bicarbonate (Sigma-Aldrich, St. Louis, Missouri), hypoxanthine (Sigma-Aldrich), gentamycin (Sigma-Aldrich), and 10% heat-treated human O+ serum (Interstate Blood Bank, Inc.) as described previously [15]. Parasite cultures were synchronized with 5% d-sorbitol (Sigma-Aldrich) two times prior to harvesting at ring stage (> 99%), and the parasitaemia of the entire culture without fractionation was determined by microscopic analysis. Native culture material was frozen for long-term storage at  −80 °C and used for preparing serial dilutions into uninfected fresh blood (PlasmaLab International, Everett, Washington). The ITG culture stock was reported to have a concentration of 10.2 ng/mL HRP2, diluted in negative whole blood to 500 and 250 pg/mL HRP2, and tested with the CELISA. The CELISA approximated the concentrations 577 and 251 pg/mL HRP2, respectively. Subsequent ITG culture dilutions were prepared using the reported concentration from the stock.

Each sample (GST-W2, FliS-W2, cultured W2, and cultured ITG) was diluted with negative whole blood to yield six serial dilutions representing HRP2 concentrations of 250, 125, 80, 40, 20, and 10 pg/mL. Each sample concentration and the negative diluent was tested with the uRDT in five replicate by highly skilled operators in a controlled laboratory setting at PATH (Seattle, Washington). Expanded testing (75 additional replicates) was conducted for each sample near the LOD, based on the lowest concentration that was repeat reactive (at least 2 of 5 replicates), along with the concentration immediately above and below the lowest repeat reactive concentration. The final sample size of 80 was based on a statistical sampling plan (ISO 2859-1:1999). General inspection level I was selected and Sample size code J was determined by lot size (3201–10,000); Single sampling plan for normal inspection was selected, which determined the sample size of 80.

### Specificity

Specificity testing was conducted with archived frozen whole blood from 123 unique donors from malaria non-endemic settings (BioreclamationIVT, Hicksville, New York). Venipuncture NaEDTA whole blood was collected from 11 additional donors from malaria non-endemic settings (PlasmaLab International, Everett, Washington) for comparing the test with matched fresh versus frozen venipuncture whole blood samples. Each sample for specificity testing was conducted in duplicate with the uRDT according to the manufacturer’s instructions by highly skilled operators at PATH. Assay diluent only (no sample) was tested in duplicate. All tests with the specificity samples were interpreted at the recommended 20 min read time and at 1 h.

### *Plasmodium falciparum* culture strains with *pfhrp2* and *pfhrp3* gene deletions

Four *P. falciparum* strains with *pfhrp* gene deletions were selected for evaluation with the uRDT. Strains D10 (BEI Resources, Manassas, Virginia) and Dd2 (BEI Resources, Manassas, Virginia) were selected for their *pfhrp2* gene deletion, but presence of *pfhrp3* gene. Strain HB3 (US Centers for Disease Control and Prevention [CDC], Atlanta, Georgia) was selected for the presence of *pfhrp2* gene, but deletion of *pfhrp3* gene. Strain 3BD5 (National Institute of Allergy and Infectious Diseases [NIAID], Bethesda, Maryland) was selected for the absence of both *pfhrp2* and *pfhrp3* genes. The ITG strain was chosen for the presence of both *pfhrp2* and *pfhrp3*. D10, Dd2, and HB3 were cultured according to the ITG culture method previously described [15]. The *P. falciparum* 3BD5 strain was cultured as described previously [16] with minor modifications to the complete media composition: 0.22% sodium bicarbonate, 20 mg/L gentamicin, 55.56 µg/L hypoxanthine, and 27.7 mM HEPES. Additionally, the cultures were maintained at 3% hematocrit between 1 and 4% parasitaemia in complete media, and O+ erythrocytes (PlasmaLab International, Everett, Washington) were washed 3 times with PBS to remove leukocytes and platelets. The 3BD5 culture was synchronized twice with 5% d-sorbitol (Sigma-Aldrich) prior to harvesting at ring stage (> 99%), and parasitaemia was determined by microscopy. The cultures of the four aforementioned *pfhrp* gene deletion strains and also previously described ITG (a *P. falciparum* strain with both *pfhrp2* and *pfhrp3* genes) were serially diluted 2-fold in whole blood from 195 to 1.56 p/µL. Each dilution of each strain was tested in duplicate with uRDT and SD-RDT according to the manufacturer’s instructions by highly skilled operators at PATH.

### Statistical analysis

uRDT specificities against frozen archived whole blood samples from malaria negative blood donors in non-endemic settings at the 20 min and 1 h time points were calculated by 100 * [(true negatives)/(true negatives + false positives)].

## Results

### Analytical sensitivity

Benchmarking results showed slight differences in test performance among the HRP2 protein types tested. As the HRP2 was diluted and the test LOD was approached, the test lines for each of the samples and dilutions decreased in intensity. Initial testing to determine the lowest concentration with repeat reactivity resulted in rGST-W2 at 80 pg/mL (3/5), rFliS-W2 at 20 pg/mL (3/5), cultured W2 at 20 pg/mL (2/5), and cultured ITG at 40 pg/mL (2/5) (Table 1). Expanded testing was then conducted and showed that the uRDT meets the AQL acceptance criteria for rGST-W2 at 125 pg/mL, rFliS-W2 and cultured W2 at 40 pg/mL, and cultured ITG at 80 pg/mL (Table 1).

**Table 1:** uRDT analytical sensitivity with two recombinant HRP2 proteins and two native culture HRP2 proteins

Target conc. (pg/mL)	rGST-W2 (%)	rFliS-W2 (%)	Cultured W2 (%)	Cultured ITG (%)
250	5/5 (100)	5/5 (100)	5/5 (100)	5/5 (100)
125	79/80 (98.8)	5/5 (100)	5/5 (100)	5/5 (100)
80	65/80 (81.3)	5/5 (100)	5/5 (100)	79/80 (98.8)
40	22/80 (27.5)	78/80 (97.5)	77/80 (96.3)	40/80 (50.0)
20	1/5 (20.0)	51/80 (63.8)	47/80 (58.8)	9/80 (11.3)
10	0/5 (0)	9/80 (11.3)	9/80 (11.3)	0/5 (0)
0	0/5 (0)	0/5 (0)	0/5 (0)	0/5 (0)

### Time-to-results and specificity testing

uRDT specificity was 100% at 20 min with the panel of 123 archived frozen whole blood samples from unexposed blood donors from a non-endemic geographic area. At 1 h, the uRDT specificity decreased to 43.1%. No difference in test results was observed in comparing the matched fresh and frozen venipuncture whole blood (Table 2). One test for each condition gave a reactive result at the read time of 20 min, resulting in a specificity of 95.5% (Table 2). The duplicate test of each reactive result was nonreactive. Testing with buffer only yielded non-reactive test results, showing control lines but no test lines (Table 2). Interpreting the tests at 1 h increased the number of observed reactive results and uninterpretable results due to hemolysis obscuring the test line (Table 2). Excluding the uninterpretable results, the specificities of fresh and frozen venipuncture whole blood at 1 h were 29.4% (5/17) and 57.1% (12/21), respectively.

**Table 2:** uRDT time-to-results and specificity testing results

Sample type	Results (20 min)	Results (1 h)
Archived frozen whole blood (n = 123 in duplicate)	0 Reactive	140 Reactive
	246 Non-reactive	106 Non-reactive
Matched fresh venipuncture whole blood (n = 11 in duplicate)	1 Reactive 21 Non-reactive	12 Reactive 5 Non-reactive 5 Uninterpretable
Matched frozen venipuncture whole blood (n = 11 in duplicate)	1 Reactive 21 Non-reactive	9 Reactive 12 Non-reactive 1 Uninterpretable
Buffer (n = 2)	0 Reactive	1 Reactive
	2 Non-reactive	1 Non-reactive

### Cross reactivity with HRP3

A total of five *P. falciparum* strains were run on the rapid tests representing different combinations of the presence of *pfhrp2* and *pfhrp3* genes: ITG (*pfhrp2*^+^, *pfhrp3*^+^), HB3 (*pfhrp2*^+^, *pfhrp3*^−^), D10 and Dd2 (*pfhrp2*^−^, *pfhrp3*^+^), and 3BD5 (*pfhrp2*^−^, *pfhrp3*^−^). All tests yielded valid results. Strains with the *pfhrp2* gene, independent of the presence of the *pfhrp3* gene (ITG and HB3) reacted similarly with both rapid tests, detecting down to 49 p/µL with the SD-RDT and 3.13 p/µL with the uRDT (Table 3). Both strains with *pfhrp2* gene deletions only (D10 and Dd2) reacted similarly with both rapid tests, detecting down to 98 p/µL (Table 3). The 3BD5 strain with both *pfhrp2* and *pfhrp3* deletions was undetectable by both SD-RDT and uRDT at all parasitaemias (Table 3).

**Table 3:** SD-RDT and uRDT performance against different *P. falciparum* malaria strains

*P. falciparum* parasitaemia (p/µL)	ITG (*pfhrp2*^+^, *pfhrp3*^+^)		D10 (*pfhrp2*^−^, *pfhrp3*^+^)		Dd2 (*pfhrp2*^−^, *pfhrp3*^+^)		HB3 (*pfhrp2*^+^, *pfhrp3*^−^)		3BD5 (*pfhrp2*^−^, *pfhrp3*^−^)	
	SD-RDT	uRDT	SD-RDT	uRDT	SD-RDT	uRDT	SD-RDT	uRDT	SD-RDT	uRDT
195	R	R	R	R	R	R	R	R	NR	NR
98	R	R	R	R	R	R	R	R	NR	NR
49	R	R	NR	NR	NR	NR	R	R	NR	NR
25	NR	R	NR	NR	NR	NR	NR	R	NR	NR
12.5	NR	R	NR	NR	NR	NR	NR	R	NR	NR
6.25	NR	R	NR	NR	NR	NR	NR	R	NR	NR
3.13	NR	R	NR	NR	NR	NR	NR	R	NR	NR
1.56	NR	NR	NR	NR	NR	NR	NR	NR	NR	NR
0	NR	NR	NR	NR	NR	NR	NR	NR	NR	NR

*R* reactive specimen, *NR* non-reactive specimen

## Discussion

The shift from high to low *P. falciparum* transmission in many endemic areas has revealed a considerable presence of asymptomatic infections that are undetectable by current field methods [2, 3, 6, 12, 17, 18]. As a result, there is a need for a more sensitive field-based diagnostic test to detect the subpatent reservoir of transmission and support effective elimination programmes [2, 6]. In this study, the analytical performance of a novel uRDT against recombinant and native HRP2 strains and archived clinical whole blood specimens was evaluated in a laboratory setting.

Evaluation of the uRDT with recombinant and cultured proteins from multiple sources according to a statistically significant sample size provides an in-depth characterization of test performance and a better description of test-to-test variability. The variability in detecting different sources of HRP2 (recombinant, culture, and different culture strains) could be due to several factors. These factors could include protein variation (e.g., number of epitope repeats), affinity of the antigen-binding fragment in the RDT for different variants of HRP2, variations in actual protein present in the sample based on the starting concentration and dilution required, and degradation of the HRP2 protein due to handling and storage [19, 20]. The LODs for rGST-W2 and rFliS-W2 HRP2 in uRDT were 125 and  40 pg/mL respectively. Similarly, the LODs for cultured *P. falciparum* W2 and ITG were 40 and  80 pg/mL HRP2 respectively, but the extent of cross-reaction by HRP3 was not explored. Across different *P. falciparum* malaria strains, the uRDT was positive as low as 3.13 p/µL, but it is important to note that there can be significant variation in HRP2 concentrations and parasitaemia in both laboratory and field conditions, and that the data reported here reflect analytical performance only. Overall, these results are reflective of those also observed when running the uRDT on clinical specimens as previously described [21]. This represents an approximate 10-fold lower limit of detection than current best-in-class RDTs, which have an approximate limit of detection for HRP2 of 800–1000 pg/mL [22].

The uRDT exhibited excellent specificity against archived *P. falciparum* negative whole blood specimens from donors in non-endemic areas. However, there were a number of false reactive and uninterpretable results at 1 h, indicating that it is critical for the tests to be interpreted at 20 min as described in the product instructions. Additionally, in the field, individuals who are parasite negative, but HRP2 positive due to persistence are detectable by commercial RDTs [23], making it essential to assess uRDT performance in endemic areas. Recently, the uRDT was tested using whole blood specimens from asymptomatic study participants in Myanmar, a low transmission area with parasitaemia and HRP2 ranging from 0.2 to 136.9 p/µL and 31.2 to 265.6 pg/mL, respectively, and Uganda, a high transmission area with parasitaemia and HRP2 ranging from 0.01 to 235,095 p/µL and 6.1 to 14.600 pg/mL, respectively [21]. When compared to qRT-PCR reference assay results, uRDT sensitivities were 44% in Myanmar and 84% in Uganda. The specificities were 99.8 and 92%, respectively, for the same two sites. In comparison, by SD-RDT, the sensitivities were 0% in Myanmar and 62% in Uganda, and the specificities were 100% and 95%, respectively. The results suggest that in different transmission settings, where parasitaemias and HRP2 distributions vary, the improvement in detection of asymptomatic infections by uRDT versus SD-RDT is inconsistent. Also, a limitation of both this study and the recently published evaluation with clinical specimens from Myanmar and Uganda is that the uRDTs were run in laboratory conditions with expert readers, which characterized baseline performance, but does not necessarily depict performance under field conditions. Thus, additional uRDT studies should be conducted in the field to further define performance.

In a limited evaluation of the uRDT and SD-RDT using different *P. falciparum pfhrp2* and/or *pfhrp3* gene deletion culture strains, the uRDT showed at least a 10-fold lower parasitaemia LOD than the SD-RDT. Additionally, both the uRDT and SD-RDT appear to have the same threshold cross-reactivity with HRP3, which has been previously reported with different HRP2-specific monoclonal antibodies and distinct parasite isolates [20]. Future studies should include evaluation of the uRDT in areas with *P. falciparum pfhrp2* and/or *pfhrp3* gene deletions.

## Conclusions

The evidence from this evaluation of the uRDT indicates that it is more sensitive than a currently available malaria HRP2 rapid test. Because the uRDT has the same form-factor and usability features as current RDTs, it can be easily adopted in low resource settings and detect a higher proportion of people with low density *P. falciparum* infections. This may help identify active case detection strategies that will successfully reduce the parasite reservoir. Accordingly, future studies should perform uRDT testing in malaria endemic areas. Concerns about overtreatment, incorrect diagnosis of febrile patients, and increased burden to malaria control programmes due to identification of more malaria cases may be valid. Further research is also needed to better understand how a more sensitive test can be implemented appropriately in a manner that is cost-effective. This initial evidence suggests that the uRDT may be an effective new tool for malaria control and elimination programmes.

## Abbreviations

ACT: artemisinin-based combination therapy
EIR: entomological inoculation rate
FAb: fragment antibody
HRP2: histidine-rich protein 2
IPTp: intermittent preventive treatment in pregnancy
LDH: lactate dehydrogenase
LOD: limit of detection
MDA: mass drug administration
p/µL: parasites per microlitre
*pfhrp2*: *Plasmodium falciparum* histidine-rich protein 2 gene
*pfhrp3*: *Plasmodium falciparum* histidine-rich protein 3 gene
pg/mL: picogram per milliliter
RDT: rapid diagnostic test
SD-RDT: Standard Diagnostics, Inc. BIOLINE Malaria Ag P.f RDT
uRDT: ultra-sensitive Alere™ Malaria Ag P.f RDT
