# Correction to: Performance of an ultra-sensitive *Plasmodium falciparum* HRP2-based rapid diagnostic test with recombinant HRP2, culture parasites, and archived whole blood samples

**DOI:** 10.1186/s12936-019-2669-2

**Published:** 2019-02-04

**Authors:** Smita Das, Roger B. Peck, Rebecca Barney, Ihn Kyung Jang, Maria Kahn, Meilin Zhu, Gonzalo J. Domingo

**Affiliations:** Diagnostics Program, PATH, Seattle, WA USA

## Correction to: Malar J (2018) 17:118 10.1186/s12936-018-2268-7

Following publication of the original article [1], the authors flagged an error concerning a reference to a product in the Methods section.

The article states “Diluent lot number: 05BDDA145, Manufacture date: 2015.02.25”.

However, it should read: “Diluent lot number: 05BDDA145, Manufacture date: **2016**.02.25”.

The author apologizes for this error.
